# Gastrointestinal and Psychiatric Symptoms Among Children and Adolescents With Autism Spectrum Disorder

**DOI:** 10.3389/fpsyt.2018.00515

**Published:** 2018-10-22

**Authors:** Emily Neuhaus, Raphael A. Bernier, See Wan Tham, Sara J. Webb

**Affiliations:** ^1^Center on Child Health, Behavior, and Development, Seattle Children's Research Institute, Seattle, WA, United States; ^2^Autism Center, Seattle Children's Hospital, Seattle, WA, United States; ^3^Department of Psychiatry and Behavioral Sciences, University of Washington, Seattle, WA, United States; ^4^Seattle Children's Hospital, University of Washington School of Medicine, Seattle, WA, United States

**Keywords:** autism, gastrointestinal, comorbidity, internalizing, externalizing, self-injury

## Abstract

Individuals with autism spectrum disorder (ASD) are at heightened risk of psychiatric comorbidities across the lifespan, including elevated rates of internalizing, externalizing, and self-injurious behaviors. Identification of medical comorbidities that contribute to these concerns may elucidate mechanisms through which psychiatric concerns arise, as well as offer additional avenues for intervention. Gastrointestinal (GI) conditions are of particular interest, as they are prevalent among those with ASD, may share genetic or neurobiological etiologies with the core features of ASD, and are linked with psychiatric difficulties in the general population. In this paper, we draw on data from nearly 2,800 children and adolescents with ASD within the Simons Simplex Collection to characterize the unique contributions of (1) autism symptoms, (2) psychosocial factors (child's age, sex, verbal and nonverbal IQ, adaptive behavior, race, and household income), and (3) GI concerns with respect to multiple psychiatric outcomes. Multiple regression models revealed unique contributions of ASD symptoms and multiple psychosocial factors such as verbal IQ, adaptive behavior, and family income to internalizing, externalizing, and self-injurious behavior. In general, higher levels of psychiatric symptoms were associated with more ASD symptoms, higher verbal IQ, lower adaptive behavior skills, and lower family income. Furthermore, levels of GI symptoms accounted for unique variance in psychiatric outcomes over and above these other factors, linking increased GI problems with increased psychiatric symptoms in children with ASD. Taken together, results indicate that the presence and quantity of GI symptoms should be considered when evaluating psychiatric and behavioral concerns among children with ASD, and that treatment of GI conditions may be an important component in alleviating a broad array of mental health concerns in this group.

## Introduction

Despite their absence from diagnostic criteria, internalizing and externalizing symptoms are frequent and pervasive among individuals with autism spectrum disorder (ASD). As early as toddlerhood, children later diagnosed with ASD evidence diminished positive affect and heightened negative affect relative to children without ASD (1, 2). During childhood, rates of anxiety, depression, aggression, and self-injurious behaviors are elevated relative to peers without ASD (3–5), and psychiatric symptoms often persist into adulthood (6). Compounding these symptoms, significant internalizing and externalizing difficulties often occur concurrently (7), with long lasting consequences with regard to quality of life, educational and vocational outcomes, and clinical service utilization throughout the lifespan (8).

Along with significant psychiatric symptoms, ASD is often characterized by a number of medical comorbidities, including seizure disorders (9), sleep difficulties (10), metabolic concerns (11), and immune system dysfunction (11). Rates of these concerns exceed not only those observed in the general population (11), but also those observed among individuals with other neurodevelopmental diagnoses such as ADHD (12). Furthermore, reviews of medical records indicate that medical comorbidities may cluster together among individuals with ASD (13), such that experiencing one concern (e.g., gastrointestinal disorder) can indicate a heightened likelihood of additional concerns [e.g., sleep disorders; (10, 14)].

Among medical comorbidities, gastrointestinal symptoms are particularly prominent among individuals with ASD, occurring nearly four times as frequently as comparison groups without ASD (15). Prevalence rates for GI concerns in ASD vary considerably depending on sample characteristics and methodological approach (16). Lower estimates place the frequency of broadly defined GI symptoms around 15–20% [e.g., (17)], whereas other researchers estimate that up to 90% of children with ASD may experience significant GI difficulties (18, 19). Differences in data collection strategies likely account, in part, for this sizeable range, as parent questionnaires appear to yield higher prevalence rates [e.g., (19)] in comparison to direct evaluation by a medical provider [e.g., 17]. Within the broader construct of GI concerns, specific symptoms reported by parents and other caregivers often include abdominal pain, chronic constipation, frequent vomiting, and gastro-esophageal reflux [see 16 for review]. Similar variability characterizes prevalence rates and relative distributions of specific symptoms, with differing findings as to the most prevalent GI symptom in ASD [e.g., constipation (17), diarrhea (19)]. Regardless of this variability, it is clear that GI difficulties affect an appreciable proportion of individuals with ASD, and such symptoms likely have considerable effects on children's educational participation, family functioning, and quality of life.

The cause(s) of such pervasive GI concerns in ASD are not fully understood, but several pathways are plausible. First, it may be that shared genetic substrates underlie both ASD and GI dysfunction, at least for some individuals with ASD. Increasingly understood to stem from complex genetic bases, ASD has thus far been associated with familial and *de novo* genetic events across hundreds of genes (20), many of which contribute to GI function as well. For example, mutations and polymorphisms in genes such as CHD8 and MET have been associated with ASD phenotypes with comorbid GI complaints [CHD8, Bernier et al. (21); MET, Campbell et al. (22)]. Downstream, comorbidity between GI concerns and ASD may reflect in part the core sensory-related symptoms often observed among affected individuals (23). For example, many children with ASD experience heightened awareness of their sensory experiences, including tactile and vestibular sensations, likely amplifying subjective experiences of GI discomfort.

The cumulative data to date support the co-occurring relationship between psychiatric and GI concerns in children with ASD. As early as preschool age, children with ASD with significant GI symptoms demonstrate higher levels of internalizing, aggressive, and repetitive behavior, with positive correlations between GI and behavioral symptoms (24). Among older children and adolescents with ASD, increased GI symptoms appear to be associated with general affective problems (25), as well as with more specific psychiatric symptoms such as anxiety (26), depression (27), irritability (26), and self-injurious behavior (28). Thus, links between GI and psychiatric concerns span both internalizing and externalizing spectrums.

To some extent, these findings echo relations observed in the general population, as children without ASD also tend to display increased GI concerns in the context of both externalizing disorders such as ADHD (29) as well as internalizing symptoms such as anxiety and depression (30). However, interactions between psychiatric and GI symptoms are particularly pertinent in ASD, as the core symptoms of ASD appear to affect these associations. Compulsive and repetitive behaviors, key diagnostic criteria for ASD, correlate with GI symptoms among children and adolescents with ASD (31). Similarly, sensory over-responsivity and anxiety provide unique contributions in the prediction of GI concerns among children and adolescents with ASD (32). Finally, core impairments in communication appear to relate to the presence and expression of GI concerns. Not only do poor expressive language skills predict nearly 12-fold increase in risk for particular GI symptoms (33), but Buie et al. (16) suggest that “problem behavior in patients with ASDs may be the primary or sole symptom of the underlying medical condition, including some gastrointestinal disorder” (p. S1).

Our overarching goal in this paper was to explore the role of gastrointestinal concerns as they relate to psychiatric symptoms among children and adolescents with ASD. Within this goal, we pursued two aims. First, we sought to document the prevalence and variety of GI concerns within a large, well-characterized sample of children and adolescents with ASD. Second, we sought to understand relationships between ASD symptoms and GI concerns over and above the effects of psychosocial factors. Given the prevalence and pervasiveness of psychiatric and gastrointestinal comorbidities in ASD, better understanding of the relations between these components may offer additional avenues for intervention for children and adults with ASD, as well as inform our understanding of the multisystemic nature and mechanisms of ASD.

## Method

### Participants

Data for the current analyses were obtained as part of the Simons Simplex Collection [SSC; (34)], a collaboration across 12 research sites within the United States in which families participated in comprehensive and rigorous phenotypic assessment. In total, the SSC includes nearly 3,000 families with exactly one child with ASD between the ages of 4 and 18 years. Following protocols approved by each site's human subjects division, research reliable clinicians evaluated children using the Autism Diagnostic Observation Schedule [ADOS; (35)] with revised algorithm scoring (36), Autism Diagnostic Interview–Revised [ADI-R; (37)], and expert clinical judgment. All participating children and adolescents met diagnostic criteria established by the Collaborative Programs of Excellence in Autism [CPEA; (38)] for autism spectrum disorder. The sample had a mean calibrated severity score of 7.44 (*SD* = 1.7; Range = 4–10) according to the guidelines developed by Gotham et al. (39), in which scores can range from 1 to 10 and scores 4 or higher fall above the diagnostic threshold. In addition, children and their parents were carefully screened for a wide range of medical and familial factors. Exclusionary criteria included the following: any family history of suspected or diagnosed ASD; a nonverbal mental age below 18 months; presence of known genetic conditions, neurological disease, or head injury; significant sensory or motor impairment; extensive perinatal complications; gestational age below 36 weeks at birth; birth weight below 2,000 g; and a primary language other than English.

The resulting sample included 2,756 children (13.6% female, 86.4% male) with the following parent-reported racial/ethnic backgrounds: African American (4.0%), Asian (4.0%), Native American or Hawaiian (0.3%), more than one race (7.8%), other than listed (4.5%), and white (78.6%). Table 1 displays descriptive statistics for the families included in the current analyses.

**Table 1:** Demographic and clinical characteristics for participants.

	**Mean (SD)**	**Range**
Age (years)	9.03 (3.6)	4–18
ADI-R Current Behavior total score	27.47 (10.1)	1–60
ADOS Calibrated Severity Score	7.44 (1.7)	4–10
Verbal IQ standard score	78.04 (31.3)	5–167
Nonverbal IQ standard score	84.52 (26.2)	9–161
Vineland-2 Composite standard score	73.13 (12.1)	27–115
CBCL Anxious/Depressed T-Score	58.36 (8.9)	50–98
CBCL Externalizing T-Score	56.58 (10.6)	32–97
RBS-R Self Injurious	2.09 (2.9)	0–21

### Measures

#### Autism spectrum disorder symptoms

The presence and degree of symptoms associated with ASD were assessed using the total score from the age-appropriate ADI-R Current Behavior algorithm, consistent with the procedure described in Neuhaus et al. (40). This approach yields a single total score that summarizes parent-report of both social-communication and restricted/repetitive domains of symptoms at the time of data collection.

#### Gastrointestinal concerns

Parents completed extensive interviews regarding the child's medical history, including the presence/absence of 7 distinct GI symptoms. Symptoms were considered to be present if they were recurrent, not attributed to known acute illnesses (e.g., food poisoning), and caused “significant bother” for the family. We computed a summary variable tallying the number of symptoms reported for each child with ASD: constipation, diarrhea, severe abdominal pain, gastro-esophageal reflux, vomiting, excessive gas, and bloating. We included data of reported symptoms that were present beyond the age of 36 months, in order to exclude symptoms (e.g., physiologic reflux) that may be benign during infancy and very early childhood, with expected resolution over time.

#### Psychiatric symptoms

We identified three psychiatric symptom clusters of interest. Parent-reported internalizing and externalizing symptoms were measured via the age-appropriate versions of the Child Behavior Checklist [CBCL; (41)]. Internalizing symptoms were indexed with T-scores from the Anxious/Depressed subscale, which contains items relating to worries, fear, sadness, and negative cognitions and self-image, without items related to somatic symptoms to avoid inflating associations with our GI measure. Externalizing symptoms were reflected in the Externalizing Problems broadband T-score, which incorporates items relating to aggression and rule-breaking. In addition to these, the Self-Injurious Behavior subscale of the Repetitive Behavior Scale- Revised [RBS-R; (42)] was used as a measure of self-directed injury (e.g., biting self, hitting self with objects). As noted earlier, symptoms in all of these areas are elevated among individuals with ASD.

#### Psychosocial characteristics

A number of child and family characteristics relevant to the emergence of psychiatric symptoms were extracted from the background and clinical information available in the SSC dataset. These included child age, biological sex, verbal and nonverbal IQ scores as assessed with age-appropriate standardized measures (43–45), child's adaptive behavior composite score (46), the family's annual household income (dichotomized as below/above $80,000), and the child's race/ethnicity (sample demographics permitted analysis of African American, Asian, and white backgrounds).

### Analytic approach

In order to understand relations between child/family factors and psychiatric outcomes, we assessed direct contributions of these factors to each of our three psychiatric symptom areas (internalizing, externalizing, self-injurious behavior). Within the two age groups corresponding to ADI-R scoring algorithms (4 through 9 years of age; 10+ years of age), we used SPSS version 19 to create separate three-level multiple regression models predicting each psychiatric measure. Within each model, missing data were handled with pairwise deletion.

- At Level 1 of the models, we entered ASD symptoms to quantify the variance in psychiatric outcomes attributable to core features of ASD.
- At Level 2, we entered psychosocial factors hypothesized to account for additional variance in psychiatric concerns. These consisted of child's age, child's biological sex, child's verbal and nonverbal IQ scores, child's adaptive behavior, family's annual income, and child's race (African American, Asian, or white).
- At Level 3, we entered GI concerns present after the age of 36 months to assess contribution of these concerns over and above ASD symptoms and psychosocial factors.

## Results

### Prevalence of parent-report GI concerns

Consistent with previous literature, families in the SSC frequently reported that their child with ASD had significant GI symptoms. Over one third of the sample (37.7%) experienced at least one symptom, with a mean of 0.61 (*SD* = 0.98; Range = 0–6) conditions endorsed. In all, 23.7% of participants experienced one GI symptom, 8.3% experienced two symptoms, 3.0% experienced three symptoms, and 2.6% experienced four or more symptoms. Table 2 provides rates of specific parent-reported GI concerns. Rates of symptoms ranged from 4.2% (vomiting) up to 24.1% (constipation) of the SSC sample. As shown, the most frequently reported symptoms were constipation, diarrhea, and excessive gas.

**Table 2:** Prevalence of parent-reported GI concerns.

	**Endorsed beyond 36 months of age**
Bloating	4.6%
Constipation	24.1%
Diarrhea	10.6%
Excessive Gas	6.9%
Reflux	5.5%
Severe Abdominal Pain	5.1%
Vomiting	4.2%

### Factors associated with psychiatric outcomes

#### Internalizing symptoms

The sample as a whole had a mean Anxious/Depressed T-score of 58.36 (*SD* = 8.9, Range = 50.0–98.0), with 24.7% of participants' scores falling at or above 65, indicating borderline or clinical level of concern. Within the younger group (ages 4:0 to 9:11), ASD symptoms did not account for significant variance in internalizing symptoms when entered alone [*F*_(1, 1681)_ = 0.25, *p* = 0.62]. The addition of psychosocial factors accounted for an additional 15.7% of the variance in internalizing, a significant increase over ASD symptoms alone [*F*_(9, 1672)_ = 35.80, *p* < 0.001]. GI symptoms accounted for 1.0% of variance over and above autism symptoms and psychosocial factors [*F*_(1, 1671)_ = 17.94, *p* < 0.001], such that the combined model accounted for 16.5% of the variance in internalizing symptoms among the younger age group [*F*_(11, 1682)_ = 31.24, *p* < 0.001]. Within the combined model, there were unique contributions from ASD symptoms, child age, verbal IQ, adaptive behavior, family income, and GI symptoms. For younger children, higher levels of internalizing symptoms were associated with higher levels of ASD symptoms, older age, higher verbal IQ, lower adaptive behavior, lower family income, and more GI concerns. See Table 3.

**Table 3:** Unique contributions of child and family factors to internalizing symptoms.

	**Younger age group (4 years < 10 years)**		**Older age group (10 yrs < 18 years)**	
	**β**	**t**	**β**	**t**
ASD symptoms	0.11	4.24^***^	0.13	3.68^***^
Age	0.24	10.71^***^	−0.01	−0.18
Child sex	−0.04	−1.57	0.04	1.38
Verbal IQ	0.43	10.06^***^	0.41	6.64^***^
Nonverbal IQ	−0.06	−1.55	−0.01	−0.21
Adaptive behavior	−0.09	−2.57^*^	−0.03	−0.68
Household income	−0.09	−4.07^***^	−0.07	−2.26^**^
**RACE/ETHNICITY**				
African American	−0.03	−1.04	−0.04	−1.26
Asian	−0.02	−0.77	−0.05	−1.47
White	−0.04	−1.61	0.05	1.26
GI symptoms	0.10	4.24^***^	0.10	3.83^***^

* *p < 0.05*.

** *p < 0.01*.

*** *p < 0.001. Internalizing symptoms assessed via Child Behavior Checklist Anxious/Depressed subscale T-scores*.

Among the older children and adolescents (ages 10:0 and older), ASD symptoms entered alone did not account for significant variance [*F*_(1, 907)_ = 1.17, *p* = 0.28]. However, psychosocial factors contributed 14.9% of variance [*F*_(9, 898)_ = 17.52, *p* < 0.001], and GI concerns accounted for an additional 1.1% [*F*_(1, 897)_ = 11.45, *p* < 0.001]. As a whole, the combined model accounted for 15.1% of the variance in internalizing symptoms [*F*_(11, 908)_ = 15.67, *p* < 0.001], with unique contributions from ASD symptoms, verbal IQ, family income, and GI symptoms. Again, higher levels of internalizing symptoms were associated with more ASD symptoms, higher verbal IQ, lower family income, and more GI symptoms.

#### Externalizing symptoms

A similar set of findings emerged with respect to externalizing outcomes. The sample as a whole had a mean Externalizing T-score of 56.6 (*SD* = 10.6, Range = 32.0–97.0), with 22.8% of participants' scores falling at or above 65. Within the younger age group, significant variance was accounted for at each level of the regression model, with unique variance accounted for by ASD symptoms [3.8% of variance, *F*_(1, 1681)_ = 68.13, *p* < 0.001], psychosocial factors [6.0% of variance, *F*_(9, 1672)_ = 12.34, *p* < 0.001], and GI concerns [1% of variance, *F*_(1, 1671)_ = 16.42, *p* < 0.001]. In total, the combined model accounted for 10.2% of the variance in externalizing symptoms [*F*_(11, 1682)_ = 18.31, *p* < 0.001]. As shown in Table 4, there were unique contributions from ASD symptoms, verbal IQ, adaptive behavior, family income, and GI symptoms. Externalizing behavior was higher when children had higher levels of ASD symptoms, higher verbal IQ, lower adaptive behavior, lower income, and more GI concerns.

**Table 4:** Unique contributions of child and family factors to externalizing symptoms.

	**Younger age group**		**Older age group**	
	**(4 years < 10 years)**		**(10 years < 18 years)**	
	**β**	**t**	**β**	**t**
ASD symptoms	0.12	4.55^***^	0.20	5.54^***^
Age	0.02	−0.98	−0.17	−5.37^***^
Child sex	0.00	0.14	0.06	1.95
Verbal IQ	0.32	7.19^***^	0.45	7.30^***^
Nonverbal IQ	−0.07	−1.55	−0.22	−3.59^***^
Adaptive behavior	−0.28	−7.46^***^	−0.18	−3.56^***^
Household income	−0.11	−4.65^***^	−0.11	−3.51^***^
**RACE/ETHNICITY**				
African American	−0.01	−0.56	−0.03	−0.70
Asian	−0.04	−1.37	0.02	0.50
White	−0.03	−0.92	0.10	2.62^**^
GI symptoms	0.09	4.05^***^	0.12	3.83^***^

** *p < 0.01*.

*** *p < 0.001. Externalizing symptoms assessed via Child Behavior Checklist Externalizing T-scores*.

For older children and adolescents, there were again significant contributions from each level of the model [ASD symptoms: 5.3% of variance, *F*_(1, 908)_ = 52.35, *p* < 0.001; Psychosocial factors: 9.7% of variance, *F*_(9, 899)_ = 11.44, *p* < 0.001; GI symptoms: 1.4% of variance, *F*_(1, 898)_ = 14.64, *p* < 0.001], and the combined model accounted for 15.5% of the variance in externalizing symptoms [*F*_(11, 909)_ = 16.16, *p* < 0.001]. However, the relative contributions of individual predictors within the combined model were somewhat different, with significant contributions from ASD symptoms, child's age, verbal IQ, nonverbal IQ, adaptive behavior, family income, child's ethnicity, and GI symptoms. Externalizing behavior was higher when children were younger, and had higher levels of ASD symptoms, higher verbal IQ, lower nonverbal IQ, lower adaptive behavior, lower income, parent-reported white ethnicity, and more GI concerns.

#### Self-injurious behavior

Among the younger children in the sample, the regression model predicting self-injurious behaviors revealed significant unique variance associated with each level of the model, including ASD symptoms alone [4.6%, *F*_(1, 1681)_ = 82.89, *p* < 0.001], psychosocial factors [4.4%, *F*_(9, 1672)_ = 9.02, *p* < 0.001], and GI concerns [1.1%, *F*_(1, 1671)_ = 21.31, *p* < 0.001]. In total, the combined model accounted for 9.7% of the variance in self-injurious behaviors [*F*_(11, 1682)_ = 17.36, *p* < 0.001]. Unique contributions were associated with ASD symptoms, adaptive behavior, family income, and GI concerns. Self-injurious behaviors were higher for children with more ASD symptoms, lower adaptive behavior, lower income, and more GI concerns. See Table 5.

**Table 5:** Unique contributions of child and family factors to self-injurious symptoms.

	**Younger age group (4 years < 10 years)**		**Older age group (10 years < 18 years)**	
	**β**	**t**	**β**	**t**
ASD symptoms	0.13	4.59^***^	0.17	4.65^***^
Age	0.04	1.52	−0.04	−1.20
Child sex	0.00	0.00	0.06	1.82
Verbal IQ	0.03	0.66	0.09	1.42
Nonverbal IQ	0.00	0.08	−0.16	−2.54^*^
Adaptive behavior	−0.18	−4.75^***^	−0.09	−1.66
Household income	−0.12	−5.20^***^	−0.07	−2.18^*^
**RACE/ETHNICITY**				
African American	−0.01	−0.30	−0.08	−2.15^*^
Asian	−0.04	−1.69	−0.03	−0.80
White	−0.02	−0.84	−0.03	−0.71
GI symptoms	0.11	4.62^***^	0.04	1.39

* *p < 0.05*.

*** *p < 0.001. Self-injurious behavior assessed via the Self-Injurious Behavior subscale of the Repetitive Behavior Scale- Revised*.

Finally, for the older children and adolescents, the regression model predicting self-injurious behavior revealed a more distinctive set of findings. As before, ASD symptoms and psychosocial factors each accounted for unique variance [ASD: 6.3%, *F*_(1, 909)_ = 60.73, *p* < 0.001; psychosocial: 3.6%, *F*_(9, 900)_ = 3.94, *p* < 0.001]. However, the addition of GI symptoms at the third level of the model did not account for significant variance over and above these [*F*_(1, 899)_ = 1.94, *p* = 0.16]. Taken together, the combined model accounted for 8.9% of the variance in self-injurious behavior [*F*_(11, 910)_ = 9.09, *p* < 0.001], with significant contributions from ASD symptoms, nonverbal IQ, family income, and child race. Older children and adolescents displayed higher levels of self-injurious behavior when they had higher levels of ASD symptoms, lower nonverbal IQ, lower income, and did not identify as African American. Unlike the models described to this point, the presence of GI concerns did not appear to contribute to level of self-injurious behaviors by parent report.

## Discussion

Our findings underscore the intertwined roles of psychiatric and gastrointestinal symptoms among children and adolescents with ASD. Although individuals with ASD are at elevated risk for both psychiatric and medical comorbidities across the lifespan (3, 5, 47), these constructs have not been fully investigated in tandem despite increasing recognition of ASD as a disorder with implications across neurobiological symptoms. In this paper, we found evidence of unique variance associated with GI symptoms across all three measures of psychiatric symptoms we examined, including internalizing, externalizing, and self-injurious behaviors. In all but one of the regression models described above, the inclusion of parent-reported GI symptoms significantly increased the statistical variance explained in psychiatric outcomes, over and above the contributions of psychosocial variables.

Beyond GI concerns, our analyses also identified a number of other child and family factors that corresponded to increased psychiatric symptoms in this sample. For both internalizing and externalizing symptoms, we found increased psychiatric difficulties when children had more ASD symptoms, higher verbal IQ scores, lower adaptive behavior skills, and lower household income. These findings fit with previous literature linking increased internalizing concerns with stronger cognitive skills (48, 49) and lower family income (50) in ASD. Patterns of findings were more distinct for older participants (age 10 years and older) with regard to externalizing and self-injurious behaviors. For both of these outcomes in our older participants, nonverbal IQ and race/ethnicity emerged as significant predictors. Specifically, higher levels of externalizing and self-injurious behavior were both associated with having a lower nonverbal IQ. Higher levels of externalizing were associated with being younger and being of white race/ethnicity, whereas lower levels of self-injurious behavior were associated with African American race/ethnicity. This overall picture—in which similar predictors hold for younger children across psychiatric outcomes while differences emerge for older children across psychiatric outcomes—might suggest a developmental trend in which psychiatric difficulties earlier in life are related to a common set of factors, whereas contributing factors begin to diverge and demonstrate more specificity in their links to outcomes as children move toward adolescence.

With regard to the prevalence of GI symptoms among individuals with ASD, our findings suggest marked GI symptoms among approximately one third of children and adolescents in this sample. As discussed earlier, estimates of prevalence span a wide range among published studies on GI function in ASD, likely due to methodological differences between them (16). Higher estimates of GI concerns may be associated with data collection approaches such as questionnaire measures and medical record review, whereas medical history data in the Simons Simplex Collection result from a standardized parent interview. In addition, participants in the SSC met strict and tightly defined diagnostic criteria for ASD, and this procedure may have excluded some children who would have been included in community-based clinic samples. As we discuss in more detail later, the SSC is unique in its recruitment purely of simplex families, and this feature likely also affects the phenotype described here.

Taken together, our findings have implications for assessment and intervention across disciplines. With regard to mental health providers, our findings indicate a need to assess for GI symptoms even when those are not the presenting complaint, as they may serve to contribute to the behavioral concerns for which a family is seeking services. Although GI symptoms accounted for relatively small proportions of variance in psychiatric symptoms in our analyses, they were nonetheless significantly associated with mental/behavioral health. Moreover, effects of GI symptoms were not limited to a single psychiatric symptom area but rather applied across three different measures. As such, despite the limited variance in some models, appropriate treatment of GI symptoms may be an important part of reducing a broad array of mental health symptomatology in this population, and may be critical in improving quality of life and overall functioning. Such recommendations are bolstered by observations that the presence of significant GI conditions among children with ASD may moderate response to psychopharmacological treatment for behavior problems (51).

Conversely, with respect to medical providers, our findings suggest that families seeking treatment for GI symptoms may benefit from a comprehensive assessment of possible “downstream” behavioral or psychological effects. Use of brief, standardized, broad-based questionnaire measures such as those included in the analyses presented here [e.g., Child Behavior Checklist; (41)] can provide medical providers with a broad overview of a child's well-being, and can assist in exploration as to whether medical treatment should be augmented with behavioral intervention (e.g., parent support, psychotherapy) to address effects in those domains.

### Limitations of the current study

As always, conclusions from the current findings should be considered within the context of the sample and measures with which they were observed. By design, the Simons Simplex Collection comprises children and adolescents with ASD with minimal familial, perinatal, or historical risk factors for autism, with the goal of enriching possible *de novo* genetic contributions to ASD (34). Given this approach, it may be that our results are most applicable to individuals with ASD who have similar familial and genetic backgrounds (i.e., simplex status), and may be less applicable to individuals with ASD in the context of positive family history for ASD, significant perinatal complications, or other identified ASD risk factors. While many occurrences of ASD do appear to be spontaneous or idiopathic in nature (34), the current approach does leave a proportion of individuals with ASD for whom the SSC may not be fully representative and for whom prevalence of GI concerns and relations between GI and psychiatric symptoms may be different than observed here.

Similarly, given the nature and goals of the SSC sample and procedures, we cannot speak to the generalizability of our findings to a broader population of individuals without ASD, such as those with other neurodevelopmental or psychiatric diagnoses. For instance, individuals with intellectual disabilities may also experience heightened prevalence of psychiatric comorbidities and gastrointestinal concerns when compared to the general population (52), but our results cannot clarify whether links between those psychiatric and GI symptoms parallel those observed in our findings. The same is true with regard to links between psychiatric and GI symptoms for children in the general population, for whom psychosocial and environmental factors are associated with GI symptoms [e.g., (53)], as our analyses do not include data from typically-developing children and adolescents.

With regard to measurement, the measures used in the current study carry both advantages and limitations. Our measures of GI (parent-reported symptoms present after the age of 36 months) and psychiatric symptoms (standardized parent-report questionnaires) rely upon parent report. A medical evaluation based on standard diagnostic criteria (ROME IV; DSM 5) and comprehensive testing (e.g., imaging, GI studies, direct assessment) may determine the etiology of symptoms, and extend our understanding of potential mechanisms underlying the gastroenterology-psychiatry relationship. Our approach also cannot clarify whether the GI concerns assessed are reported by caregivers with equal reliability, as some may be more apparent to parents (e.g., vomiting) while others (e.g., nausea, pain) may yield fewer observable signs and rely more on children's ability to understand and communicate their internal state. In addition, the nature of our GI variable does not include evaluation of the severity and impact on quality of life. Severity, rather than quantity, of GI symptoms may have greater impact on psychiatric well-being, and symptom severity may show stronger associations between measures of GI and psychiatric health. For example, a child with severe abdominal pain may experience greater negative impact, with reduced participation in physical activities and increased school absenteeism, compared to a child with multiple but less severe GI symptoms who may continue in their normative roles. The summary variable in the current study cannot capture the severity of GI concerns, and such questions will be important aspects for future work. Finally, as GI symptoms are aggregated into a composite score, we cannot identify whether and how specific GI concerns have more fine-grained or unique associations with particular psychiatric symptoms.

Despite these considerations, this approach likely mirrors typical clinical situations encountered by medical and mental health providers, in which families present for medical or psychiatric issues and clinicians must rely upon parent report or questionnaire methods to gain information, with limited access to prior diagnostic evaluations and/or referrals to specialty evaluation. It is also promising to note that parents' reports of children's GI symptoms tend to be relatively strong indicators of true GI conditions (33), suggesting that the GI variables included in the SSC dataset stand as a reasonable proxy for these purposes.

### Future directions

Moving forward, conclusions from our analyses and others [e.g., (24, 25, 27)] regarding the links between psychiatric and GI comorbidities among individuals with ASD would be strengthened by use of longitudinal data that could inform questions of causality and the direction of effects between symptoms. Our findings are consistent with interpretations positing a causal influence such that the presence of GI symptoms are associated with increased likelihood of mental health symptoms, either as a result of ongoing physical discomfort and/or as a behavioral expression of that discomfort. However, while these interpretations are both plausible and consistent with previous thinking [e.g., (16)], the current data cannot confirm that direction of effect due to the cross-sectional nature of the SSC dataset.

Future research should also explore how comorbidities between ASD, behavioral, and medical (including GI) concerns relate to the biological mechanisms and genetic substrates of ASD. A number of candidate genes in which *de novo* changes can increase likelihood of ASD also appear to carry effects on GI function, both in people with ASD and in animal models of analogous genetic changes. For example, protein truncating mutations to *CHD8*, a chromatin remodeling gene expressed both in the central and enteric nervous systems, are among the most common disruptive mutations identified in ASD through exome sequencing (21). What is more, these *de novo* changes also correspond to significant GI dysfunction (primarily constipation) for 83% of affected individuals with ASD, and to disrupted gut motility in zebrafish models of those same *de novo* changes (21). Delineation of mechanistic pathways through which genetic events result in an ASD phenotype will be critical, and better understanding of intertwined medical and psychiatric comorbidities may suggest candidate systems in those pathways as well as identify additional systems influencing phenotype (e.g., regulation of serotonin, implicated in both gut and brain function).

Together, findings presented here reinforce the need for conceptualizing ASD as a diagnosis affecting multiple neurobiological systems. With this perspective comes the potential of (1) identifying comprehensive research questions to clarify ASD etiology and mechanism, (2) exploring meaningful subgroups within the larger ASD diagnosis to guide that research, and (3) offering multiple points of intervention through which to support affected individuals and their families. In light of the current findings, ties between psychiatric and gastrointestinal comorbidities may be particularly well-suited for these purposes and will continue to be an important avenue for investigation.

## Ethics statement

This study was carried out in accordance with the recommendations of the University of Washington Human Subjects Committee with written informed consent from all subjects. All subjects gave written informed consent in accordance with the Declaration of Helsinki. The protocol was approved by the University of Washington Human Subjects Committee.

## Author contributions

The authors made substantial contributions to the data collection (RB), conception and design (EN, SW, and ST), and analysis (EN). All authors participated in interpretation of the data and manuscript drafting, revising, and approval.

### Conflict of interest statement

The authors declare that the research was conducted in the absence of any commercial or financial relationships that could be construed as a potential conflict of interest.
